# Availability and performance of image/video-based vital signs monitoring methods: a systematic review protocol

**DOI:** 10.1186/s13643-017-0615-3

**Published:** 2017-10-25

**Authors:** Mirae Harford, Jacqueline Catherall, Stephen Gerry, Duncan Young, Peter Watkinson

**Affiliations:** 1 Kadoorie Centre for Critical Care and Trauma Research and Education, John Radcliffe Hospital, Headley Way, Oxford, OX3 9DU UK; 2 Nuffield Department of Clinical Neurosciences, University of Oxford, Oxford, UK; 3 Centre for Statistics in Medicine, University of Oxford, Oxford, UK

**Keywords:** Monitor, Non-invasive, Non-contact, Image, Camera, Vital signs

## Abstract

**Background:**

For many vital signs, monitoring methods require contact with the patient and/or are invasive in nature. There is increasing interest in developing still and video image-guided monitoring methods that are non-contact and non-invasive. We will undertake a systematic review of still and video image-based monitoring methods.

**Methods:**

We will perform searches in multiple databases which include MEDLINE, Embase, CINAHL, Cochrane library, IEEE Xplore and ACM Digital Library. We will use OpenGrey and Google searches to access unpublished or commercial data. We will not use language or publication date restrictions. The primary goal is to summarise current image-based vital signs monitoring methods, limited to heart rate, respiratory rate, oxygen saturations and blood pressure. Of particular interest will be the effectiveness of image-based methods compared to reference devices. Other outcomes of interest include the quality of the method comparison studies with respect to published reporting guidelines, any limitations of non-contact non-invasive technology and application in different populations.

**Discussion:**

To the best of our knowledge, this is the first systematic review of image-based non-contact methods of vital signs monitoring. Synthesis of currently available technology will facilitate future research in this highly topical area.

**Systematic review registration:**

PROSPERO CRD42016029167

**Electronic supplementary material:**

The online version of this article (10.1186/s13643-017-0615-3) contains supplementary material, which is available to authorized users.

## Background

Monitoring of the basic functions of the human body is routine practice in healthcare settings from primary care to critical care. The term ‘vital signs’ typically refers to measurements including heart rate (HR), respiratory rate (RR), blood pressure (BP), oxygen saturations (SpO_2_) and temperature. Vital signs monitoring methods such as the electrocardiograph (ECG) and pulse oximetry have the advantages of being easily available, quick and non-invasive. In many clinical settings, these widely available monitors may be sufficient. However, they do have disadvantages which may limit their use in high dependency areas.

Examples of their limitations include the following:

- ECG monitoring relies on application of three or more ECG electrodes on the patient. These consist of conducting gel surrounded by an adhesive area. Signal acquisition relies on good contact between the gel and the patient’s skin and this process is prone to errors from electrostatic and electromagnetic interference and skin impedance. The small amplitude bioelectrical signals measured are prone to interference from other biological signals or machinery.
- A single point of measurement may limit the amount of information being collected [1]. A broader receiving system may offer more detailed spatial information simultaneously from multiple sites, allowing derivation/mapping of physiological parameters. Ultimately, this may facilitate insights that would be difficult to appreciate from a single-point analysis.
- There are infection risks from being physically attached to monitors and the use of the same machines on multiple patients.
- Monitors that require contact with the skin may lead to skin barrier breakdown. This is a concern in critical care patients who are more prone to hospital-acquired pressure ulcers and impaired skin healing [2], especially in neonates [3].
- Multiple lines for various forms of monitoring create a hazard for both patients and staff, particularly during transfer. They may also interfere with clinical care.
- Multiple contact monitors can interfere with sleep which is known to adversely affect recovery [4].
- Non-contact monitoring is likely to be less emotionally traumatic for visitors in critical care settings [5].

Some of these limitations and complications may be avoided by using non-contact, non-invasive monitoring methods in the form of image/video-based monitoring. There is a huge potential for this type of monitoring in the current era of telemedicine, and the required technology has developed rapidly in the last decade. Several image-based technologies have been explored for vital signs monitoring, and it is our aim to provide a summary of their current availability and performance.

## Objective

We aim to review non-contact, non-invasive image/video-based vital signs monitoring devices in health care and health-related fields. To our knowledge, this will be the first systematic review of the evidence for this type of monitoring. The review will provide a basis for further research using similar technology in a rapidly evolving field.

## Methods/design

### Protocol and registration

Methods of the systematic review have been developed in accordance with the Preferred Reporting Items for Systematic Reviews and Meta-Analyses (PRISMA) [6] guideline. The completed PRISMA-P [7] checklist is available as a supplementary file to this protocol (Additional file 1).

This proposed systematic review has been registered with PROSPERO (the International prospective register of systematic reviews): CRD42016029167.

### Eligibility criteria

All papers looking at monitoring of HR, BP, RR or SpO_2_ using image analysis with comparison to a clinically validated reference device will be included without limitation on the setting. We will exclude temperature monitoring which lends itself to infrared imaging and has been extensively studied [8–10]. Technology which require placement of trackers or monitors on the body will be excluded. Studies using projected markers but require image/video analysis for monitoring purposes will be included. No limitations will be set on the primary objective of the studies; for example, studies aiming to refine image analysis will be included as long as they measure one of the specified vital signs and compare it to a clinically validated reference device as part of the methodology.

Initial screening process will select any literature reviews to ensure all original studies are included. Only human subject studies will be included, and no age limits will be set. Date and language restrictions will not be applied. Authors of studies published in languages other than English will be contacted by email for assistance with data extraction.

All unpublished studies found and articles in the media will be included wherever possible to minimise publication bias.

We will include randomised controlled trials, interventional studies, observational studies (including case-control or cohort studies) and pilot studies that compare an image-based monitoring system to any established forms of monitoring already used in clinical practice. Only studies that compare results from an image methodology with a clinically validated reference non-image methodology will be included. Any case series or reports will be included in the first instance with no limitations on the total number of subjects.

### Outcomes

The primary intended outcome is a summary of the current availability of image/video-based vital signs monitoring methods and a comparison of the performance of the new technology with other clinically available methods.

Secondary outcomes of interest are the quality of the studies’ methodology with respect to the Guidelines for Reporting Reliability and Agreement Studies (GRRAS) [11], current limitations in image/video-based monitoring technology and the feasibility of its use in different populations.

It is probable that there will be considerable variability in the outcome measurements and the reporting of the technology’s efficacy. We will aim to produce a qualitative synthesis if the heterogeneity between the studies does not allow a quantitative summary.

### Data sources/management

Searches will be performed in multiple databases including MEDLINE, Embase, CINAHL and Cochrane library. Given the subject topic, IEEE (Institute of Electrical and Electronics Engineers) Xplore Digital Library and ACM (Association for Computing Machinery) Digital Library will also be searched. OpenGrey will be searched for any unpublished grey literature. Given the popular culture/commercial applications of the technology, Google/Google Scholar will also be searched.

If any websites are available, primary original research data from which the technology is based will be sought.

Identified references will be downloaded to reference library software for the initial title and abstract screening. All references proceeding to the next stage will be handled using Distiller SR software (Evidence Partners, Ottawa, Canada). All searches will be saved for referencing, including a full list of papers and timeline of searches. The process of each paper will be clearly documented from the screening and review process.

### Search strategy

The search strategy will be guided by a medical librarian who will advise on the conduct of the searches and limitations of the search terms used. Broad search terms will be used in order to capture all publications on this topic.

Initial search terms of interest include Monitor/Invasive/Non-invasive/Contact/Non-contact/Contactless/Remote/Image/Camera/Webcam/Web-cam/Physiologic/Measure/Respiratory rate/Breathing rate/Pulse/Heart rate/Blood pressure/SpO2.

Where appropriate, the terms will be exploded and MeSH (Medical Subject Headings) terms will be used. As demonstrated in the example, word stems will be used to generate results where appropriate.

Once searches are complete, the findings will be pooled and any duplicates removed.

Draft search strategy is outlined in Table 1.

**Table 1:** Example of search strategy

1	(remote*).ab,ti.
2	(measur*).ab,ti.
3	(imag*).ab,ti.
4	(monitor*).ab,ti.
5	(camera* OR webcam* OR web-cam* OR video).ab,ti.
6	(invasive* OR non-invasive* OR noninvasive OR contact* OR non-contact* OR noncontact* OR remote*).ab,ti.
7	(respira* OR respiratory rate OR breathing rate OR pulse OR heart rate OR blood pressure OR SpO2).ab,ti.
8	1 AND 2 AND 5
9	2 AND 3 AND 6 AND 7
10	4 AND 6 AND 7
11	5 AND 6
12	“Monitoring, Physiologic/methods”[MAJR]
13	5 AND 12
14	6 AND 12
15	8 OR 9 OR 10 OR 11 OR 13 OR 14

*Word truncation search

### Study selection and data extraction

A four-stage review process will be used to select final articles for inclusion.

Stage 1: Once searches are generated and checked for duplicates, the titles will be screened by two reviewers (MH, JC) to reject any which are clearly unrelated. Any articles with uncertainty or where there is a disagreement between the reviewers will be included for abstract review.
Stage 2: Abstract review will be undertaken by two reviewers (MH, JC). Where there is a discrepancy, the article will proceed to a full-text review along with others selected by both reviewers.
Stage 3: A selection of full-text articles will be assessed by two reviewers (MH, JC) using a pilot data extraction form on the reference management software. The extraction output will be compared and a final data extraction form will be created. All full texts will then be assessed by the two reviewers. Authors will be contacted by email for clarification or for further data wherever necessary.
Stage 4: Once articles are selected, hand-searching strategy will be used to review their references to look for relevant articles not already identified. This is of particular importance as pseudo-healthcare use of non-contact monitoring or non-published articles may be identified using this method.

The returned papers will be categorised under the predominant technology used. Anticipated headings include the following:

- Non-contact photoplethysmography (PPG)/imaging PPG
- Thermal/infrared
- Multi-slice image and tracking
- Tracking of projected light

The above categories will be included in the data extraction form and effort will be made to group similar technologies together for analysis. We expect to stratify studies by predominant imaging modality, vital signs of interest and the target population.

### Outcomes and prioritisation

The following data will be extracted from each study: the type and date of publication, funding source, conflict of interest, study type, vital sign of interest, setting, the number of participants, population and cohort data, eligibility criteria, image technology used, reference method, distance from camera to subject, body part imaged, frames per second, simulated physiological changes, performance of new technology compared to reference method, limitations reported, statistical analysis method, main conclusions and data to allow quality of reporting assessment according to GRRAS guidelines.

The primary intended outcome is a qualitative review of the availability and performance of different technology used for each vital sign. The studies will be categorised according to the video/image-based monitoring used (e.g. PPG, thermal) and the vital sign being measured (i.e. HR, BP, RR, SpO_2_) during the data extraction phase.

A secondary intended outcome is an appraisal of the quality of reporting among the method comparison studies included in the review with respect to the modified GRASS scoring system (Table 2). Furthermore, a summary of the current limitations of non-contact, non-invasive technology, potential methods of improving image acquisition and the application of the technology in different clinical populations will be synthesised.

**Table 2:** Adapted GRRAS tool for quality assessment of measurement comparison studies

Section	Original guidelines from GRRAS	Scoring	
Title and abstract	Identify in title or abstract that interrater/intrarater reliability or agreement was investigated	N/A^a^	
Introduction	Name and describe the diagnostic or measurement device of interest explicitly	The image-based vital signs monitor is described	+
		No description	–
	Specify the subject population of interest	Specifies the subject population of interest	+
		No population specified	–
	Specify the rater population of interest (if applicable)	N/A	
	Describe what is already known about reliability/agreement and provide a rationale for the study	Describes what is already known about the reliability of image-based monitoring method and provide a rationale for the study	+
		No statement on the current knowledge of the method and no rationale stated	–
Methods	Explain how the sample size was chosen. State the determined number of raters/subjects/objects/replicate observations	Explains how the sample size was chosen and/or state the determined number of subjects/replicate observations^b^	+
		No explanation of sample size. Number of subjects/replicate observations not stated	–
	Describe sampling method	Describes sampling method	+
		No description of sampling method	–
	Describe the measurement/rating process (e.g. time interval between repeated measurements, availability of clinical information, blinding)	Describes the measurement process	+
		No description of measurement process	–
	State whether measurements/ratings were conducted independently	Two (or more) methods of measurements conducted independently	+
		Measurements not conducted independently	–
	Describe the statistical analysis	Describes the statistical analysis planned	+
		No description of statistical analysis planned	–
Results	State the actual number of raters and subjects/objects which were included and the number of replicate observations which were conducted	States the actual number of subjects who were included and number of observations (e.g. duration of recording)	+
		No statement of the number of subjects/observations	–
	Describe the sample characteristics of raters/subjects	N/A	
	Report estimates of reliability and agreement including measure of statistical uncertainty	Reports estimates of reliability and agreement including measure of statistical uncertainty using gold standard measures	+
		Reports estimates of reliability and agreement including measure of statistical uncertainty using acceptable standard measures	?
		Reports estimates of reliability and agreement including measure of statistical uncertainty but using inappropriate measures/no estimates of reliability/agreement given	–
Discussion	Discuss the practical relevance of results	Discusses the practical relevance of results	+
		No discussion of practical relevance of results	–
Auxillary material	Provide detailed results if possible (e.g. online)	N/A	

*N/A* not applicable

^a^Studies will not be penalised for not stating the gold standard/reference method of monitoring in the title/abstract as we aim to include studies where this comparison was performed with an alternative primary aim (e.g. improve image analysis protocol)

^b^Pilot studies will not be penalised for not stating how the sample size was chosen

### Assessment of study quality

The appraisal will be performed by two reviewers (MH, JC) who will agree on the final assessment. In the event of a discrepancy, this will be resolved by discussion with a third reviewer (PW or DY).

Many studies of interest for the purpose of this review are likely to be experimental/pilot/proof-of-concept studies using a small number of subjects from a single centre. It is anticipated that it may not be possible to weight studies numerically in the final analysis due to the likely variability in the study designs and variable study sizes.

GRRAS recommendation for method comparison studies reporting is designed to cover a broad range of clinical test scores/classification/diagnosis and not all applicable for our use. Therefore, they have been adapted to suit our objectives (Table 2).

A key aspect of quality assessment is the appropriateness of the statistical analysis. Pearson’s correlation coefficient is not an appropriate statistical method to assess reliability and agreement, as originally discussed by Bland and Altman [12]. Despite this, a review of statistical methods used to test for agreement of medical instruments showed that the correlation coefficient remains the second most frequently used statistic [13]. In order to assess this aspect of each study, the statistical analysis will be checked against the published method agreement analysis guidelines [14]. The classification strategy based on the guidelines is summarised in Table 3. Each study will be assessed for statistical appropriateness. This has been incorporated to the modified GRRAS scoring system (Table 2).

**Table 3:** Classification of statistical methods used to compare image-guided vital signs measurement against reference device

Gold standard	Acceptable	Inappropriate
• Bland-Altman plot/limits of agreement (LOA) analysis • Intraclass correlation coefficient • Lin’s concordance correlation coefficient • British standards reproducibility/repeatability coefficient For all methods in this category, if repeat measurements per patient have not obviously been accounted for, then classify as acceptable.	• Mean square error/deviation • Root mean square error/deviation • Mean absolute error/deviation • Accuracy • Categorisation (e.g. proportion of time/measurements test within a threshold of gold standard) • Descriptive only—plot of test against gold standard, or test and gold standard signals overlaid	• (Pearson’s) correlation coefficient • *T* test

Where one method is used, or all methods used are in the same category, the overall rating will be given as that category. Where multiple methods are used and they are in adjacent categories, the study will be categorised as the better. If gold standard and inappropriate methods are used together, the study will be classified as acceptable.

It is expected that Bland and Altman’s method will be used most frequently for measuring agreement. Specifically, it is important that where multiple observations are made per individual (e.g. multiple HR measurements from one subject using two methods), this is accounted for in the statistical assessment [15]. This has been incorporated into the statistical rating scale (Table 3).

Each included study will be given a rating based on the modified GRASS scoring system as below:

- All items are “+”: high quality
- All items are “+” except the statistical analysis rating of “?” OR one of the other sections is given “-”: moderate quality
- Statistical analysis is given “-” OR two or more sections are given “-”: low quality

Finally, the GRADE methodology will be used to systematically rate the strength of the overall review as high/moderate/low/very low [16].

### Heterogeneity

There is likely to be a high degree of heterogeneity across the studies given the likely variation in reported outcomes. It is hoped that it would be possible to compare methods post stratification by the vital signs of interest (e.g. HR) and by the predominant technology used.

### Missing data

We will seek missing data by contacting original authors. Effort will be made to include all available studies.

### Subgroup analysis

Subgroup analysis will be undertaken for the group of studies with acceptable statistical methods and outcomes summarised in numerical format if possible. Should the extracted data be deemed sufficiently homogenous from a clinical standpoint for quantitative synthesis to be undertaken, we anticipate performing numerical analysis. If we identify studies that are sufficiently homogenous, we will attempt a meta-analysis of the results, using random-effects methods to combine measures of bias and agreement from multiple Bland-Altman analyses [17]. Similar techniques may also be used to combine measures of accuracy and error. If significant heterogeneity exists between the types of study making the numerical subgroup analysis impossible, the analysis will be qualitative. If there is sufficient stratified data for the application or performance of any particular imaging modality or detected vital sign, a statistical subgroup analysis will be performed.

### Amendments to protocol

Any deviation from this protocol will be dated and documented to ensure a transparent review process. As per PRISMA guidelines, no changes will be made to the main body of the protocol and any unanticipated additional findings will be discussed in the final review.

## Discussion

This systematic review will synthesise a summary of the current technology available for image-based monitoring of vital signs.

The main strength of the proposed review is that a summary of the availability and effectiveness of non-invasive, non-contact monitoring methods and their utility in the clinical environment will provide a basis for the direction of future work in the field. Furthermore, an appraisal of the quality of reporting among these studies will guide authors for reporting of future studies. Similarly, a review of the statistical methods employed among these studies will improve the analysis of future studies.

### Limitations

Despite the outlined wide search strategy, we anticipate a number of difficulties in carrying out the proposed systematic review. There is high likelihood of publication bias as the published studies/imaging methods are more likely to have better performance compared to those that are not published. We also anticipate that studies conducted as part of doctoral theses may not be found despite the wide search strategy and/or may be difficult to access. It is likely that a proportion of these studies will have been published in peer-reviewed journals with further likelihood of publication bias. Every effort will be made to include grey literature available with review of citations/references and advice from a librarian.

We also anticipate that a large number of studies included in the review will be proof-of-concept studies with very few subjects. Therefore, we anticipate a heterogeneous group of studies to be included in the review which may make quantitative summary difficult.

We believe that this review will provide a useful summary of the current technologies available for image-based monitoring of vital signs and guide the direction of future research for more complex monitoring, especially in critical care settings.

## Additional file

Additional file 1: PRISMA-P. (DOCX 21 kb)

## Abbreviations

BP: Blood pressure
ECG: Electrocardiograph
GRRAS: Guidelines for Reporting Reliability and Agreement Studies
HR: Heart rate
ICU: Intensive care unit
LOA: Limits of agreement
PPG: Photoplethysmography
PRISMA: Preferred Reporting Items for Systematic Reviews and Meta-Analyses
RR: Respiratory rate
SpO_2_: Oxygen saturations

## References

[1] Sun Y, Papin C, Azorin-Peris V, Kalawsky R, Greenwald S, Hu S (2012). Use of ambient light in remote photoplethysmographic systems: comparison between a high-performance camera and a low-cost webcam. J Biomed Opt.

[2] Mestha LK, Kyal S, Xu B, Lewis LE, Kumar V. Towards continuous monitoring of pulse rate in neonatal intensive care unit with a webcam. Conf Proc IEEE Eng Med Biol Soc. 2014:3817–20. 10.1109/EMBC.2014.6944455 25570823

[3] Johnson DE (2016). Extremely preterm infant skin care: a transformation of practice aimed to prevent harm. Adv Neonatal Care.

[4] Kamdar BB, Needham DM, Collop NA (2012). Sleep deprivation in critical illness: its role in physical and psychological recovery. J Intensive Care Med.

[5] Hardicre J. Meeting the needs of families of patients in intensive care units. Nurs Times 2003;99(27):26-27. 12882048

[6] Moher D, Liberati A, Tetzlaff J, Altman DG, The PRISMA Group (2009). Preferred Reporting Items for Systematic Reviews and Meta-Analyses: the PRISMA Statement. PLoS Med.

[7] Moher D, Shamseer L, Clarke M, Ghersi D, Liberati A, Petticrew M, Shekelle P, Stewart LA (2015). Preferred Reporting Items for Systematic Review and Meta-Analysis Protocols (PRISMA-P) 2015 Statement. Syst Rev.

[8] Aubakir B, Nurimbetov B, Tursynbek I, Varol HA. Vital sign monitoring using Eulerian video magnification and thermography. Conf Proc IEEE Eng Med Biol Soc. 2016:3527–30. 10.1109/EMBC.2016.7591489 28269059

[9] Wang K, Gill P, Wolstenholme J, Price CP, Heneghan C, Thompson M, Pluddemann A (2014). Non-contact infrared thermometers for measuring temperature in children: primary care diagnostic technology update. Br J Gen Pract.

[10] Lam H, Kostov Y, Tolosa L, Falk S, Rao G (2012). High resolution non-contact fluorescence based temperature sensor for neonatal care. Meas Sci Technol.

[11] Kottner J, Audige L, Brorson S, Donner A, Gajewski BJ, Hrobjartsson A, Roberts C, Shoukri M, Streiner DL (2011). Guidelines for Reporting Reliability and Agreement Studies (GRRAS) were proposed. J Clin Epid.

[12] Bland JM, Altman DG (1986). Statistical methods for assessing agreement between two methods of clinical measurement. Lancet.

[13] Zaki R, Bulgiba A, Ismail R, Ismail NA (2012). Statistical methods used to test for agreement of medical instruments measuring continuous variables in method comparison studies: a systematic review. PLoS One.

[14] Watson PF, Petrie A (2010). Method agreement analysis: a review of correct methodology. Theriogenology.

[15] Bland JM, Altman DG (2007). Agreement between methods of measurement with multiple observations per individual. J Biopharm Stat.

[16] Belsham H, Helfand M, Schunemann HJ, Oxman AD, Kunz R, Brozek J (2011). GRADE guidelines: 3. Rating the quality of evidence. J Clin Epidemiol.

[17] Tipton E, Shuster J. A framework for the meta-analysis of Bland-Altman studies based on a limits of agreement approach. Stat Med. 2017; doi: 10.1002/sim.7352. [Epub ahead of print] 10.1002/sim.7352 PMC5585060 28664537

